# Nirmatrelvir/ritonavir and risk of long COVID symptoms: a retrospective cohort study

**DOI:** 10.1038/s41598-023-46912-4

**Published:** 2023-11-11

**Authors:** Seth Congdon, Zev Narrowe, Nang Yone, Jacob Gunn, Yuting Deng, Priya Nori, Kelsie Cowman, Marjan Islam, Sharon Rikin, Joanna Starrels

**Affiliations:** 1 Department of Medicine, Montefiore Medical Center, 111 East 210th Street, Bronx, NY 10467 USA; 2 Albert Einstein College of Medicine, Bronx, NY USA; 3 Department of Critical Care Medicine, Montefiore Medical Center, Bronx, NY USA

**Keywords:** Infectious diseases, Outcomes research

## Abstract

We conducted a retrospective cohort study to assess whether treatment with nirmatrelvir/ritonavir was associated with a reduced risk of long COVID. We enrolled 500 adults with confirmed SARS-CoV-2 who were eligible for nirmatrelvir/ritonavir; 250 who took nirmatrelvir/ritonavir and 250 who did not. The primary outcome was the development of one or more of eleven prespecified long COVID symptoms, assessed through a structured telephone interview four months after the positive SARS-CoV-2 test. Multivariable logistic regression models controlled for age, sex, race/ethnicity, chronic conditions, and COVID-19 vaccination status. We found that participants who took nirmatrelvir/ritonavir were no less likely to develop long COVID symptoms, compared to those who did not take the medication (44% vs. 49.6%, *p* = 0.21). Taking nirmatrelvir/ritonavir was associated with a lower odds of two of the eleven long COVID symptoms, brain fog (OR 0.58, 95% CI 0.38–0.88) and chest pain/tightness (OR 0.51, 95% CI 0.28–0.91). Our finding that treatment with nirmatrelvir/ritonavir was not associated with a lower risk of developing long COVID is different from prior studies that obtained data only from electronic medical records.

## Introduction

Long COVID is an incompletely understood syndrome that affects millions of COVID-19 survivors^1–3^. Patients are diagnosed with long COVID when they develop or continue to experience symptoms or conditions that are not fully explained by other medical conditions four weeks or longer after acute COVID-19^4,5^. While some patients with long COVID return to baseline after a few months, many continue to experience symptoms for many months to years, often preventing them from returning to their pre-COVID-19 activities^6–9^. As of June 2023, roughly 80% of Americans experiencing long COVID reported activity limitations from their symptoms, and roughly 25% reported significant activity limitations^10^. It is estimated that long COVID is keeping millions of Americans out of the workforce^11^. Identifying tools to reduce the risk of developing long COVID or the duration of long COVID symptoms is imperative.

Several risk factors have been associated with developing long COVID^12^. The severity of acute COVID-19 has been associated with the number of long COVID symptoms experienced^13^. The level of SARS-CoV-2 viremia has been shown to be associated with acute COVID-19 symptom duration^14^, and one study found that detectable SARS-CoV-2 viremia at the time of initial infection is a risk factor for developing long COVID^15^. Nirmatrelvir/ritonavir is an oral antiviral therapy that received FDA emergency use authorization (EUA) in December 2021 for the treatment of mild to moderate COVID-19, and it has been shown to significantly reduce the risk of progression to severe COVID-19 in high risk patients^16^. It is plausible that nirmatrelvir/ritonavir may lower the risk of long COVID by inhibiting viral replication and limiting viremia during acute COVID-19. One retrospective cohort study found that nirmatrelvir/ritonavir treatment was associated with reduced risk of long COVID^17^, but it used electronic medical record (EMR) data alone, which may have not captured patients experiencing long COVID who did not seek medical care. Additionally, the study sample was mostly non-Hispanic White and included few participants from racial and ethnic groups that have been disproportionally affected by COVID-19^18,19^, limiting generalizability.

In order to investigate the impact of nirmatrelvir/ritonavir on development of long COVID in a real-world setting, we conducted a retrospective cohort study to determine the association of treatment with nirmatrelvir/ritonavir with incidence of long COVID symptoms.

## Methods

### Study design and setting

This retrospective cohort study took place from May to November 2022 at Montefiore Medical Center and Montefiore Medical Group (hereafter referred to as “Montefiore”), an academic medical center comprising three hospitals and a group of primary and specialty care clinics serving Bronx and Westchester Counties, New York. The majority of Montefiore patients live in the Bronx, which has a predominantly Hispanic and non-Hispanic Black population^20^. The study was approved by the Montefiore-Einstein Institutional Review Board (IRB), all methods were performed in accordance with the relevant guidelines/recommendations, and all study participants provided verbal informed consent.

### Participants

Inclusion and exclusion criteria were selected to ensure all study participants were eligible to be prescribed nirmatrelvir/ritonavir, and that long COVID symptoms reported were temporally related to their COVID-19 infection four months ago. The four-month timeframe was chosen as the first nirmatrelvir/ritonavir prescriptions were sent on December 29th, 2021, and this study was approved by the IRB in late April 2022. Inclusion criteria were age greater than or equal to 18 years old and meeting criteria to be prescribed nirmatrelvir/ritonavir under the Food and Drug Administration’s EUA^21,22^. Exclusion criteria were: (1) taking a medication with a clinically relevant drug-drug interaction with nirmatrelvir/ritonavir that could not be held or dose-reduced for the duration of the nirmatrelvir/ritonavir course^23^, (2) advanced kidney disease (eGFR < 30 mL/min), (3) advanced liver disease (Child–Pugh class C), (4) unable to consent and/or unable to reasonably recount symptoms accurately (e.g., patients with dementia, intellectual disability or altered mental status), (5) having a subsequent episode of COVID-19 since the infection four months prior, (6) experiencing long COVID symptoms from a prior SARS-CoV-2 infection that had not resolved fully before the infection four months prior, (7) being treated with chemotherapy in the month prior to enrollment, as it may cause symptoms which overlap with long COVID, and (8) being treated with molnupiravir, an antiviral alternative to nirmatrelvir/ritonavir with a similar mechanism of action.

The prevalence of long COVID among people who survive acute COVID-19 varies among different cohorts which have used different definitions. Population-based surveys and the largest meta-analysis to date have reported a prevalence between 6.2% and 35.1%^10,24,25^. To determine the sample size for the current study, we therefore estimated the expected prevalence to be 20%. With this assumption, enrolling 250 patients who were treated with nirmatrelvir/ritonavir and 250 patients who were not treated would yield at least 80% power at the 0.05 level of significance to detect an absolute difference in having long COVID of 10% or greater, which we deemed to be clinically significant.

### Screening and enrollment

Using the Montefiore EMR, we identified patients prescribed nirmatrelvir/ritonavir, and patients who were not prescribed nirmatrelvir/ritonavir, during the period of December 29th, 2021, going forward in time until 500 study participants were enrolled. To identify nirmatrelvir/ritonavir patients, we generated a list of all patients prescribed nirmatrelvir/ritonavir by a Montefiore medical provider. To identify control patients, we generated a list of all patients who tested positive for SARS-CoV-2 on a polymerase chain reaction (PCR) test done at Montefiore on each day patients were prescribed nirmatrelvir/ritonavir.

To screen for eligibility, we first reviewed patients’ medical records. Next, we attempted to contact potential participants via phone. We attempted to contact all patients prescribed nirmatrelvir/ritonavir. For the control patients—a much larger group—a different strategy was used: the list of patients who tested positive for SARS-CoV-2 on each nirmatrelvir/ritonavir prescription date was randomized, and going down that list in sequential order we attempted to contact potential participants until one or more were enrolled, or we reached the end of the list. Patients were considered “unable to be contacted” if they were not successfully contacted on two separate occasions, or were contacted but requested to be called back at a future time, and then did not answer the phone when called at that future time. Individuals who were successfully contacted and agreed to participate in the study were then screened for the full inclusion and exclusion criteria.

### Data collection

Participants completed a single structured telephone interview conducted by a physician or medical student four months (120 to 150 days) after their positive SARS-CoV-2 test. Participants were asked to identify which, if any, treatments they received to treat their acute COVID-19 episode four months ago. If participants were prescribed nirmatrelvir/ritonavir, they were asked if they completed a full course, partial course, or did not take it. For participants that took a partial course, they were asked to estimate the number of doses taken out of the ten total doses. Individuals were then asked to self-identify as having long COVID by responding “yes” or “no” to whether they were currently experiencing any new or worsened symptoms since developing COVID-19 four months prior. They were then asked if they were experiencing eleven common long COVID symptoms: dyspnea or change in how their breathing feels; change in smell or taste; headaches; dizziness or lightheadedness; chest pain or pressure; palpitations; generalized fatigue or tiredness; exertional intolerance; nausea/vomiting or abdominal discomfort; brain fog; and paresthesias^4,26–29^. It was emphasized that they should only answer “yes” if the symptom was either new or had worsened since developing COVID-19 four months prior and had not fully resolved. The full study survey is included in the supplemental materials. The study survey was a de novo instrument.

### Outcomes

We considered long COVID to be present if participants were experiencing any of the long COVID symptoms listed in the survey. The primary outcome was the presence of long COVID. Secondary outcomes were: the presence of each individual long COVID symptom, the number of long COVID symptoms per participant, and the number of participants who self-identified as having long COVID.

### Other variables assessed

Baseline characteristics that may influence the risk of developing long COVID^12^ were assessed through manual EMR chart review and verbal confirmation with patients. Predefined covariates considered to be potential confounders were age; race/ethnicity; sex; body mass index (BMI); COVID-19 vaccination status (primary series plus booster, primary series without booster, or unvaccinated); smoking status (current, former, never); and preexisting conditions at the time of developing COVID-19 four months prior: hypertension, diabetes, asthma or chronic obstructive pulmonary disease, or a mood disorder (anxiety disorder, depressive disorder, bipolar disorder, posttraumatic stress disorder). We also assessed the number of nirmatrelvir/ritonavir doses taken, and if individuals received prespecified treatments during their acute COVID-19 episode (monoclonal antibodies, remdesivir, corticosteroids and convalescent plasma; confirmed by EMR review).

### Statistical analysis

We used descriptive statistics to report characteristics and frequency of any and each of the long COVID symptoms in the treated and not treated groups. We used chi-square tests or t-tests to compare the demographics between the two groups. To determine whether receipt of nirmatrelvir/ritonavir was associated with long COVID symptoms, we conducted separate bivariate chi-square tests or t-tests for each of the primary and secondary outcomes, comparing between the two groups. To visualize the incidence of individual long COVID symptoms in the two groups, we created a Forest Plot presenting the odds ratio for each symptom. Finally, we conducted a series of separate multivariable logistic regression models where the main independent variable was treatment group (treated or not treated), and the dependent variable was the primary outcome. We included the following potential confounders as covariates in the model, as they have been associated with higher incidence of long COVID in prior studies: female sex, obesity (BMI ≥ 30), elderly age (≥ 65 years old), smoking history, diabetes, lung disease, mood disorder, and unvaccinated status^12^. We also included Hispanic race/ethnicity as a covariate, as compared to other racial/ethnic groups this group has reported higher rates of long COVID on the United States Census Bureau’s Long COVID Household Pulse Survey^10^.

## Results

**Figure 1:**
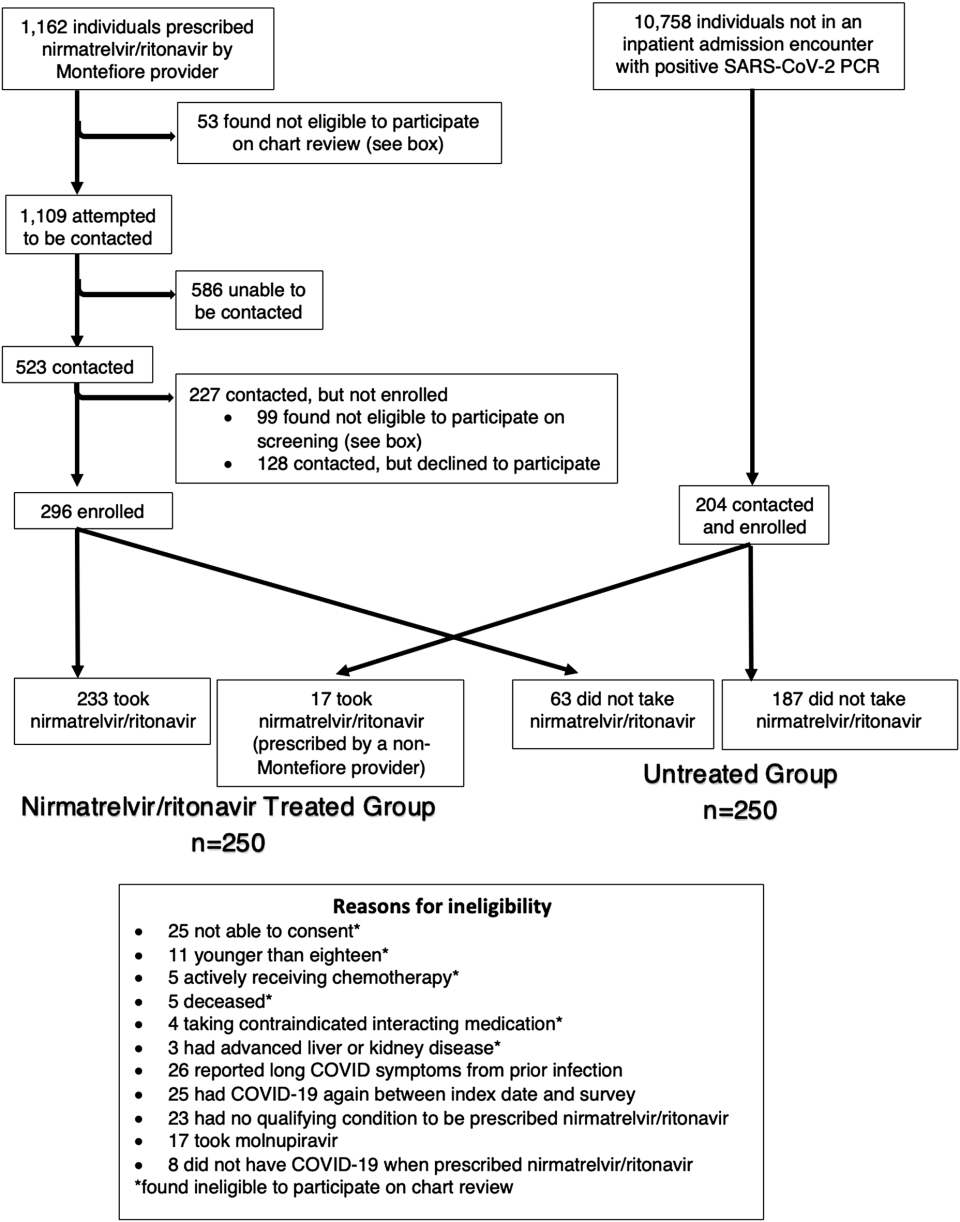
Participant flow diagram.

A total of 1162 individuals prescribed nirmatrelvir/ritonavir by a Montefiore provider were screened to reach the enrollment target. During the corresponding timeframe, a total of 10,758 individuals tested positive for SARS-CoV-2 on PCR and were eligible for screening (Fig. 1).

Participants characteristics are summarized in Table 1. The mean age was 50.6, they were 70% female, 34.4% Hispanic, 29.2% non-Hispanic White, and 26.2% non-Hispanic Black. Of the 500, five (1%) were hospitalized for acute COVID-19, and none were admitted to the intensive care unit. Compared with the untreated group, nirmatrelvir/ritonavir-treated participants were: older, more likely to be non-Hispanic White, less likely to be non-Hispanic Black or Hispanic, and more likely to have received a booster vaccination.

**Table 1:** Participant characteristics.

	Overall cohort(n = 500)	Nirmatrelvir/ritonavir treated group(n = 250)	Untreated group(n = 250)	*p*-value
Age in years, mean (std)	50.6 (15.2)	52.5 (15.1)	48.7 (15)	0.006**
Female sex, number (%)	350 (70%)	166 (66.4%)	184 (73.6%)	0.08
Race/ethnicity, number (%)				< 0.0001***
Hispanic	172 (34.4%)	71 (28.4%)	101 (40.4%)	
Non-Hispanic White	146 (29.2%)	104 (41.6%)	42 (16.8%)	
Non-Hispanic Black	131 (26.2%)	49 (19.6%)	82 (32.8%)	
Asian	41 (8.2%)	21 (8.4%)	20 (8%)	
Other/unknown	10 (2%)	5 (2%)	5 (2%)	
Hospitalized, number (%)	5 (1%)	2 (0.8%)	3 (1.2%)	1.0
Vaccination status, number (%)				< 0.0001***
Primary series + booster	338 (67.6%)	191 (76.4%)	147 (58.8%)	
Primary series alone	123 (24.6%)	39 (15.6%)	84 (33.6%)	
Unvaccinated	39 (7.8%)	20 (8%)	19 (7.6%)	
Other treatment, number (%)				0.46
Monoclonal antibody	15 (3%)	6 (2.4%)	9 (3.6%)	
Corticosteroids	8 (1.6%)	4 (1.6%)	4 (1.6%)	
Remdesivir	1 (0.2%)	1 (0.4%)	0	
Convalescent plasma	1 (0.2%)	1 (0.4%)	0	
BMI, mean (std)	30.94 (7.4)	31.44 (8.1)	30.4 (6)	0.13
Hypertension, number (%)	197 (39.4%)	103 (41.2%)	94 (37.6%)	0.41
Diabetes, number (%)	89 (17.8%)	51 (20.4%)	38 (15.2%)	0.13
Asthma or COPD, number (%)	136 (27.2%)	74 (29.6%)	62 (24.8%)	0.23
Mood disorder, number (%)	136 (27.2%)	70 (28%)	66 (26.4%)	0.69
Tobacco smoking status, number (%)				0.98
Current	44 (8.8%)	22 (8.8%)	22 (8.8%)	
Former	118 (23.6%)	60 (24%)	58 (23.2%)	
Never	338 (67.6%)	168 (67.2%)	170 (68%)	

Significant values are in [**,***].

* denotes *p* < 0.05, ** denotes *p* ≤ 0.01 and *** denotes *p* ≤ 0.001.

Table 2 summarizes the presence of long COVID symptoms between groups (primary outcome), as well as the total number of symptoms per person, the number of people in each group who self-identified as experiencing long COVID, and the presence of individual long COVID symptoms in each group. Compared to the untreated group, fewer participants who took nirmatrelvir/ritonavir had long COVID symptoms (44% vs. 50%), though the difference was not statistically significant (*p* = 0.21). The total number of symptoms per participant was also lower in the nirmatrelvir/ritonavir group (1.33 vs. 1.72; *p* = 0.05). Fewer people in the nirmatrelvir/ritonavir group self-identified as experiencing long COVID symptoms (14.8% vs. 21.2%; *p* 0.06).

**Table 2:** Long COVID Symptoms by Treatment. Group Presence of one or more long COVID symptoms (primary outcome), total number of symptoms per participant, number of participants self-identifying as having long COVID, and presence of individual long COVID symptoms are shown for the nirmatrelvir/ritonavir-treated group and untreated group.

	Nirmatrelvir/ritonavir treated group	Untreated group	*p*-value
Experiencing one or more symptoms, number (%)	110 (44%)	124 (50%)	0.21
Number of symptoms, mean (SD)	1.33 (2.01)	1.72 (2.41)	0.05
Self-identify as having long COVID, number (%)	37 (14.8%)	53 (21.2%)	0.06
Dyspnea, number (%)	25 (10%)	37 (14.8%)	0.1
Parosmia/dysgeusia, number (%)	18 (7.2%)	22 (8.8%)	0.51
Headaches, number (%)	32 (12.8%)	36 (14.4%)	0.6
Dizziness, number (%)	23 (9.2%)	29 (11.6%)	0.38
Chest pain/tightness, number (%)	19 (7.6%)	35 (14%)	0.02*
Palpitations, number (%)	21 (8.4%)	28 (11.2%)	0.29
Generalized fatigue, number (%)	58 (23.2%)	62 (24.8%)	0.68
Activity intolerance, number (%)	49 (19.6%)	55 (22%)	0.51
Nausea, vomiting or abdominal pain, number (%)	15 (6%)	18 (7.2%)	0.59
Brain fog, number (%)	49 (19.6%)	74 (29.6%)	0.01**
Paresthesias, number (%)	25 (10%)	34 (13.6%)	0.21

Significant values are in [*,**].

* denotes *p* < 0.05, ** denotes *p* ≤ 0.01 and *** denotes *p* ≤ 0.001.

**Figure 2:**
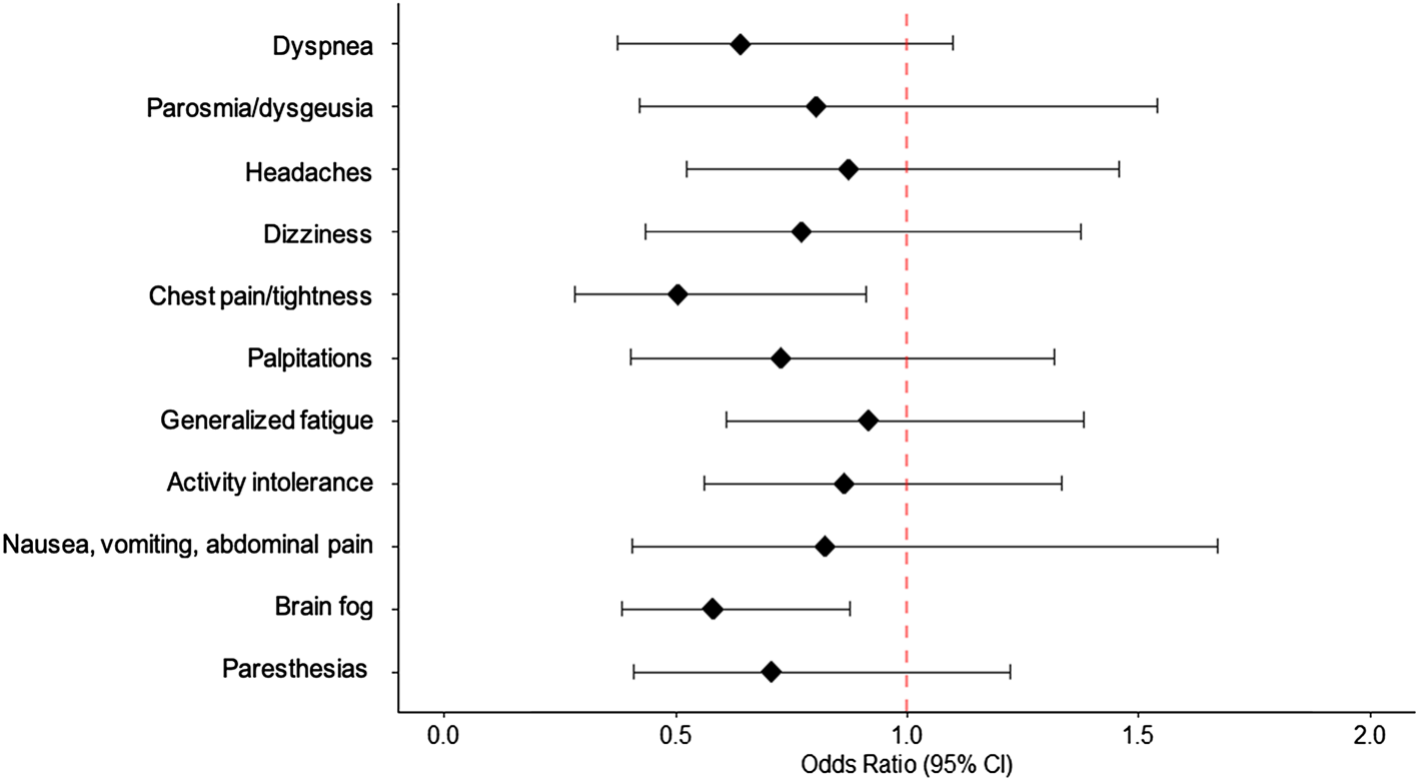
Odds ratio of individual long COVID symptoms by treatment group. Forrest plot presenting the odds of experiencing individual long COVID symptoms in patients who were treated with nirmatrelvir/ritonavir compared to those who were untreated.

Figure 2 shows the odds of individual long COVID symptoms between the nirmatrelvir/ritonavir-treated group and untreated group. Among the eleven long COVID symptoms assessed, brain fog (OR 0.58, 95% CI 0.38–0.88) and chest pain/tightness (OR 0.51, 95% CI 0.28–0.91) were lower in the nirmatrelvir/ritonavir group compared to the untreated group.

Table 3 summarizes the odds of long COVID symptoms controlling for patient characteristics. Female sex (OR 1.54, 95% CI 1.02–2.33), obesity (OR 1.47, 95% CI 1.01–2.14), history of smoking (OR 1.68, 95% CI 1.13–2.51) and history of mood disorder (OR 1.56, 95% CI 1.02–2.37) were associated with increased risk of long COVID symptoms. Age greater than 64, Hispanic race/ethnicity, diabetes, asthma/COPD, and being unvaccinated were not associated with increased risk of long COVID symptoms.

**Table 3:** Multivariate model. Results of multivariate regression analysis for presence of one or more long COVID symptoms controlling for patient characteristics.

Variable	OR	95% CI	*p*-value
Took nirmatrelvir/ritonavir (yes vs. no)	0.83	(0.57, 1.2)	0.31
Female sex (yes vs. no)	1.54	(1.02, 2.33)	0.04*
BMI > = 30 (yes vs. no)	1.47	(1.01, 2.14)	0.045*
Age > = 65 (yes vs. no)	0.72	(0.45, 1.16)	0.18
Race/Ethnicity Hispanic (yes vs no)	1.09	(0.74, 1.62)	0.66
Smoking history (yes vs. no)	1.68	(1.13, 2.51)	0.01**
Diabetes (yes vs. no)	0.83	(0.5, 1.36)	0.45
Asthma or COPD (yes vs. no)	1.19	(0.78, 1.81)	0.43
Mood disorder (yes vs. no)	1.56	(1.02, 2.37)	0.04*
Unvaccinated vs. vaccinated (primary series alone and primary series + booster)	1.1	(0.56, 2.18)	0.78

Significant values are in [*,**].

* denotes *p* < 0.05, ** denotes *p* ≤ 0.01 and *** denotes *p* ≤ 0.001.

## Discussion

This study, conducted in a real-world setting with a diverse population, found that treatment with nirmatrelvir/ritonavir was not associated with a statistically significant reduction in long COVID symptoms overall, but was associated with a statistically significant reduction in the individual symptoms of brain fog and chest pain/pressure. These findings differ from those of a study comparing 35,717 people who were prescribed nirmatrelvir/ritonavir to 246,976 controls, which reported that nirmatrelvir/ritonavir was associated with a 26% reduction in post-COVID conditions (PCC)^17^. There are several possible explanations for this. That study assessed thirteen different PCC through EMR review (International Classification of Diseases, Tenth Revision codes and laboratory results), only four of which correspond to the long COVID symptoms we assessed (dysrhythmia, fatigue/malaise, neurocognitive impairment, and shortness of breath). Our study involved direct interviews with patients, so it is possible more patients treated with nirmatrelvir/ritonavir in their study were experiencing these long COVID symptoms, but had not yet reported them to a medical provider and had it documented in the EMR as PCC. Conversely, it is possible our direct interview format could have introduced response bias, with participants responding “yes” to more symptoms than if they were administered a survey to fill out themselves. It is also possible that we overestimated the prevalence of long COVID in our study population. A study investigating rates of long COVID symptoms between individuals infected with Delta and with Omicron found that once participants reached ninety or more days post-acute infection, the proportion of individuals reporting any long COVID symptoms decreased in those infected with Omicron, but not with Delta^30^. Another study involving participants across all regions of the United States as well as Puerto Rico found that only 10% of individuals infected with Omicron and enrolled within 30 days of infection reported post-acute sequelae of SARS-CoV-2 infection (PASC) at six months^31^. We interviewed participants infected during the Omicron phase of the pandemic at a timepoint greater than 90 days out from their acute infection, so it is possible a significantly larger sample size was needed to demonstrate that nirmatrelvir/ritonavir reduces the risk of long COVID symptoms at four months into the post-acute phase.

The finding that nirmatrelvir/ritonavir caused a reduction in the individual symptoms of brain fog and chest pain/tightness is harder to explain. Multiple pathophysiologic explanations of long COVID have been proposed^32,33^, but none preferentially affect the brain, heart, or sensory peripheral nervous system. It is possible that by limiting viremia, nirmatrelvir/ritonavir lowers the risk of direct viral invasion of the myocardium and myocarditis.

We identified notable differences between the nirmatrelvir/ritonavir-treated and untreated groups. The nirmatrelvir/ritonavir treated group was overall older. This was likely because age 65 or greater is one criterion to qualify for this treatment. The nirmatrelvir/ritonavir-treated group had more non-Hispanic White participants and fewer non-Hispanic Black and Hispanic participants, as compared to the untreated group. Considering that less than 10% of the Bronx’s population is non-Hispanic White^20^, a disproportionate amount of the total nirmatrelvir/ritonavir prescriptions ordered by Montefiore providers during the study timeframe were for non-Hispanic White patients (Supplemental Table 1). This is reflective of racial and ethnic disparities in the outpatient treatment of COVID-19 in the United States as a whole^34,35^. Additionally, while a similar proportion of nirmatrelvir/ritonavir-treated and untreated participants were unvaccinated, more nirmatrelvir/ritonavir-treated participants had received a booster vaccine compared to the untreated group. Studies have shown to varying degrees that vaccination with the primary series lowers the risk of developing long COVID^36^, but the association between receiving a booster vaccine and risk of long COVID is less clear. If booster vaccination can reduce the risk of long COVID more than receiving the primary series alone, the fact that more participants treated with nirmatrelvir/ritonavir had received a booster vaccine may have exaggerated the effect of nirmatrelvir/ritonavir.

Our finding that vaccination was not associated with a decreased risk of long COVID differs from previous studies^31,36,37^. It is not clear why our findings differed, and we offer several possible explanations. While the sample size of 500 was large, it is possible that it was underpowered to see a significant difference. Study participants may have had humoral immunity from prior SARS-CoV-2 infections, which could have reduced their risk of developing long COVID. It is also possible that participants who were opposed to vaccination may have minimized and not reported symptoms.

An unexpected finding was that in both the nirmatrelvir/ritonavir-treated and untreated groups, fewer people self-identified as having long COVID than ended up reporting one or more long COVID symptoms. It is possible that participants, despite reporting the presence of such symptoms, did not perceive them to be significant. We did not assess the severity of individual symptoms or the degree to which they were impacting participants’ lives. Another possibility is that some participants’ failure to make the connection between their symptoms and long COVID is reflective of the cognitive challenges they are facing. Of the 144 participants who did not self-identify as having long COVID despite reporting long COVID symptom(s), brain fog was the most common symptom experienced (Supplemental Table 2). A meta-analysis assessing fatigue and cognitive impairment in post-acute COVID-19 patients found higher rates of cognitive impairment in studies which used objective rather than subjective modes of measurement^38^.

This study had several strengths and limitations. Study participants reflected a diverse range of ages and race/ethnicities, increasing generalizability. Study participation involved direct interviews, allowing us to confirm whether participants took nirmatrelvir/ritonavir or not, the presence or absence of long COVID symptoms participants were experiencing, and that these symptoms had developed after participants’ most recent COVID-19 episode, as opposed to persisting from a prior episode. Our study included a large number of Hispanic and non-Hispanic black participants, groups that have suffered disproportionately from the effects of the COVID-19 pandemic and have been underrepresented in research studies. Regarding limitations, participants were recruited from a single medical center, reducing generalizability. The nirmatrelvir/ritonavir-treated group was older as compared to the untreated group, potentially introducing confounding. We used a de novo survey to examine a subset of long COVID symptoms selected to cover a broad range of common symptoms without exhausting study participants and increasing recall bias. It is possible other long COVID symptoms that nirmatrelvir/ritonavir lowers the risk for were not captured; for example, we did not administer neuropsychiatric scales.

In conclusion, this retrospective cohort study found that nirmatrelvir/ritonavir was not associated with a statistically significant reduction in long COVID symptoms overall, but was associated with a statistically significant reduction in the individual symptoms of brain fog and chest pain/pressure. An optimistic interpretation of these results is that nirmatrelvir/ritonavir does reduce risk of long COVID, but our study was underpowered to detect this. It is also possible our results were those of chance, and nirmatrelvir/ritonavir does not reduce the risk of long COVID. Racial and ethnic inequalities in treatment with nirmatrelvir/ritonavir were demonstrated across the first six months of its availability, and efforts to eliminate this are vital.

## Data availability

The authors confirm that the data supporting the findings of this study are available within the article and its supplementary materials.

### Supplementary Information

Supplementary Information 1.

Supplementary Information 2.
