# Supplementary material for: Nirmatrelvir/ritonavir and risk of long COVID symptoms: a retrospective cohort study
Source: Sci Rep. 2023 Nov 11;13:19688. doi: 10.1038/s41598-023-46912-4 (PMC10640584; doi:10.1038/s41598-023-46912-4)
Supplement: Supplementary file 2 — Supplementary Information 2. [file 41598_2023_46912_MOESM2_ESM.pdf]

Title: Nirmatrelvir/ritonavir and risk of long COVID symptoms: A retrospective cohort study

Authors: Seth Congdon MD[1]*, Zev Narrowe[2], Nang Yone[2], Jacob Gunn[2], Yuting Deng[1], Priya Nori MD[1], Kelsie Cowman MPH[1], Marjan Islam MD[3], Sharon Rikin MD,MS[1], Joanna Starrels MD,MS[1]

[1]Department of Medicine, Montefiore Medical Center, Bronx, NY, United States.

[2]Albert Einstein College of Medicine, Bronx, NY, United States.

[3]Department of Critical Care Medicine, Montefiore Medical Center, Bronx, NY, United States.

Corresponding Author: Seth Congdon, MD

Department of Medicine, Montefiore Medical Center

111 East 210th Street, Bronx, NY 10467

scongdon@montefiore.org

Supplemental Table 1 Nirmatrelvir/ritonavir prescriptions ordered by Montefiore providers during the study timeframe by race/ethnicity.

Race/ethnicity	Number of prescriptions (%)
Hispanic	405 (34.9%)
Non-Hispanic White	349 (30%)
Non-Hispanic Black	274 (23.6%)
Asian	108 (9.3%)
Other/unknown	26 (2.2%)

Supplemental Table 2 Percentage of individual long COVID symptoms reported by the n = 144 participants who did not self-identify as having long COVID.

Symptom	%
Dyspnea	11%
Parosmia/dysgeusia	11%
Headaches	25.5%
Dizziness	20%
Chest pain/tightness	14.5%
Palpitations	15.9%
Generalized fatigue	42.8%
Activity intolerance	37.2%
Nausea, vomiting or abdominal pain	12.4%
Brain fog	48.3%
Paresthesias	20%

Study Survey

Date ________________ **Participant ID _______**

Was this the only time you've gotten COVID, or have you had it more than once? If more than once, were you fully recovered (felt back to normal) by the time you got COVID most recently?

Were you prescribed any medicines to treat the COVID? If yes, which ones did you take?

(If prescribed Paxlovid) Did you take the full course (2-3 pills BID x5 days)?
Full ____ Partial (# of doses out of 10) ____ Didn't take it ____
If didn't take full course, why?

Are you experiencing any new or worsened symptoms since you were diagnosed with COVID-19? Yes ___ No ___

I am going to ask you about different symptoms to see if you are or are not having them. It is important that you only answer "yes," you are having them, if these symptoms are new or worsened since you were diagnosed with COVID-19. If you are experiencing the symptom, but were already experiencing it to the same degree before you got COVID-19, answer "no."

Shortness of breath or change in how your breathing feels? Yes ___ No ___

Change in your sense of smell or taste? Yes ___ No ___

Headaches? Yes ___ No ___

Dizziness or lightheadedness? Yes ___ No ___

Chest pain or chest tightness? Yes ___ No ___

Palpitations (feeling your heart beat abnormally)? Yes ___ No ___

Generalized fatigue/tiredness? Yes ___ No ___

Exertional intolerance (getting tired when you try to do things that prior to getting COVID-19 did not make you tired)? Yes ___ No ___

Nausea, vomiting or abdominal discomfort? Yes ___ No ___

Brain fog (thinking, concentration, or memory issues)? Yes ___ No ___

Numbness, tingling or burning pain in any part of your body? Yes ___ No ___
